# Importance of the green color, absorption gradient, and spectral absorption of chloroplasts for the radiative energy balance of leaves

**DOI:** 10.1007/s10265-017-0910-z

**Published:** 2017-03-14

**Authors:** Atsushi Kume

**Affiliations:** Faculty of Agriculture, Kyushu University, 6-10-1 Hakozaki, Higashi-ku, Fukuoka 812-8581 Japan

**Keywords:** Absorption spectra, Carotenoids, Chloroplast movement, Direct radiation, Photosystem, Palisade tissue

## Abstract

Terrestrial green plants absorb photosynthetically active radiation (PAR; 400–700 nm) but do not absorb photons evenly across the PAR waveband. The spectral absorbance of photosystems and chloroplasts is lowest for green light, which occurs within the highest irradiance waveband of direct solar radiation. We demonstrate a close relationship between this phenomenon and the safe and efficient utilization of direct solar radiation in simple biophysiological models. The effects of spectral absorptance on the photon and irradiance absorption processes are evaluated using the spectra of direct and diffuse solar radiation. The radiation absorption of a leaf arises as a consequence of the absorption of chloroplasts. The photon absorption of chloroplasts is strongly dependent on the distribution of pigment concentrations and their absorbance spectra. While chloroplast movements in response to light are important mechanisms controlling PAR absorption, they are not effective for green light because chloroplasts have the lowest spectral absorptance in the waveband. With the development of palisade tissue, the incident photons per total palisade cell surface area and the absorbed photons per chloroplast decrease. The spectral absorbance of carotenoids is effective in eliminating shortwave PAR (<520 nm), which contains much of the surplus energy that is not used for photosynthesis and is dissipated as heat. The PAR absorptance of a whole leaf shows no substantial difference based on the spectra of direct or diffuse solar radiation. However, most of the near infrared radiation is unabsorbed and heat stress is greatly reduced. The incident solar radiation is too strong to be utilized for photosynthesis under the current CO_2_ concentration in the terrestrial environment. Therefore, the photon absorption of a whole leaf is efficiently regulated by photosynthetic pigments with low spectral absorptance in the highest irradiance waveband and through a combination of pigment density distribution and leaf anatomical structures.

## Introduction

Green plants absorb incident solar radiation and harness part of that energy in photosynthesis. The initial slopes of the photosynthetic light-response curves in healthy leaves are similar among a wide range of plant species, and the photosynthesis rate is proportional to the incident photon flux density of photosynthetically active radiation (PAR, 400–700 nm). However, photosynthesis curves are saturated with a high light intensity and light-saturated photosynthetic rates show large differences among leaves, even in the same individual. Furthermore, very high irradiances can damage the photosystems, and a range of mechanisms are known to bypass photoinhibition or photo-oxidation (Yamori 2016). To acclimate to changes in their environment, photosynthetic organisms have evolved direct and indirect mechanisms that respond to excess light. Several photochemical and chemical dissipation systems exist to manage the excess energy absorbed by the chloroplasts under high light conditions (Hikosaka et al. 2004; Li et al. 2009; Müller et al. 2001; Ort 2001). Leaves are arranged to avoid exposure to damaging excessive radiation (Jiang et al. 2006; Jones 2014; Muraoka et al. 1998).

Because light-use efficiency is important for understanding biomass production, several leaf photosynthesis models that take into account the light absorption profile have been proposed based on the optimal use of PAR photons (Farquhar 1989; Hikosaka and Terashima 1995; Terashima et al. 2016; Terashima and Saeki 1985). Most of the discussion has been concentrated on the efficient use of incident PAR photons in photosynthesis. However, the effects of the energy balance of chloroplasts and pigment characteristics on leaf physiological conditions have not been given close attention, although the leaf lamina’s energy balance, which determines water use in photosynthesis, has been frequently considered (Jones 2014). The effects of the spectral characteristics of incident light from the sun on the energy balance of a leaf have also not been considered.

**Fig. 1:**
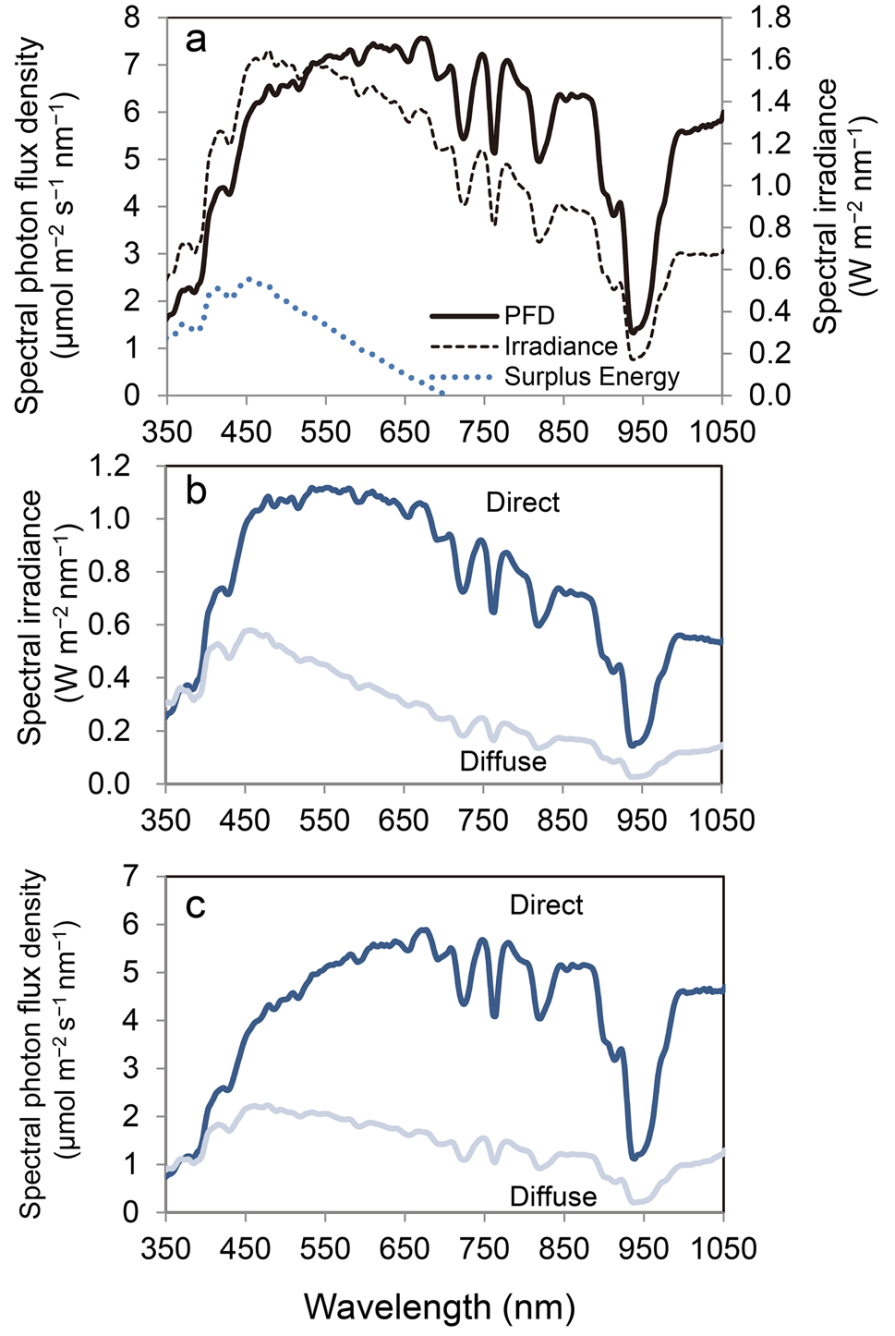
An example of spectral irradiance and photon flux density (PFD) measured on a clear day (day of year = 195) in 2011 at noon (36.05°N, 140.12°E). Measurements were conducted at 1-min intervals averaged over 1 h (11:30 am to 12:30 pm). **a** Spectral irradiance and PFD of global solar radiation. Surplus energy for photosynthesis (**Es**) is also shown (see the main text). **b** Spectral irradiance of direct (*dark line*) and diffuse radiation (*light line*). **c** Spectral PFD of direct (*dark line*) and diffuse radiation (*light line*) (Adapted from Kume et al. 2016)

**Fig. 2:**
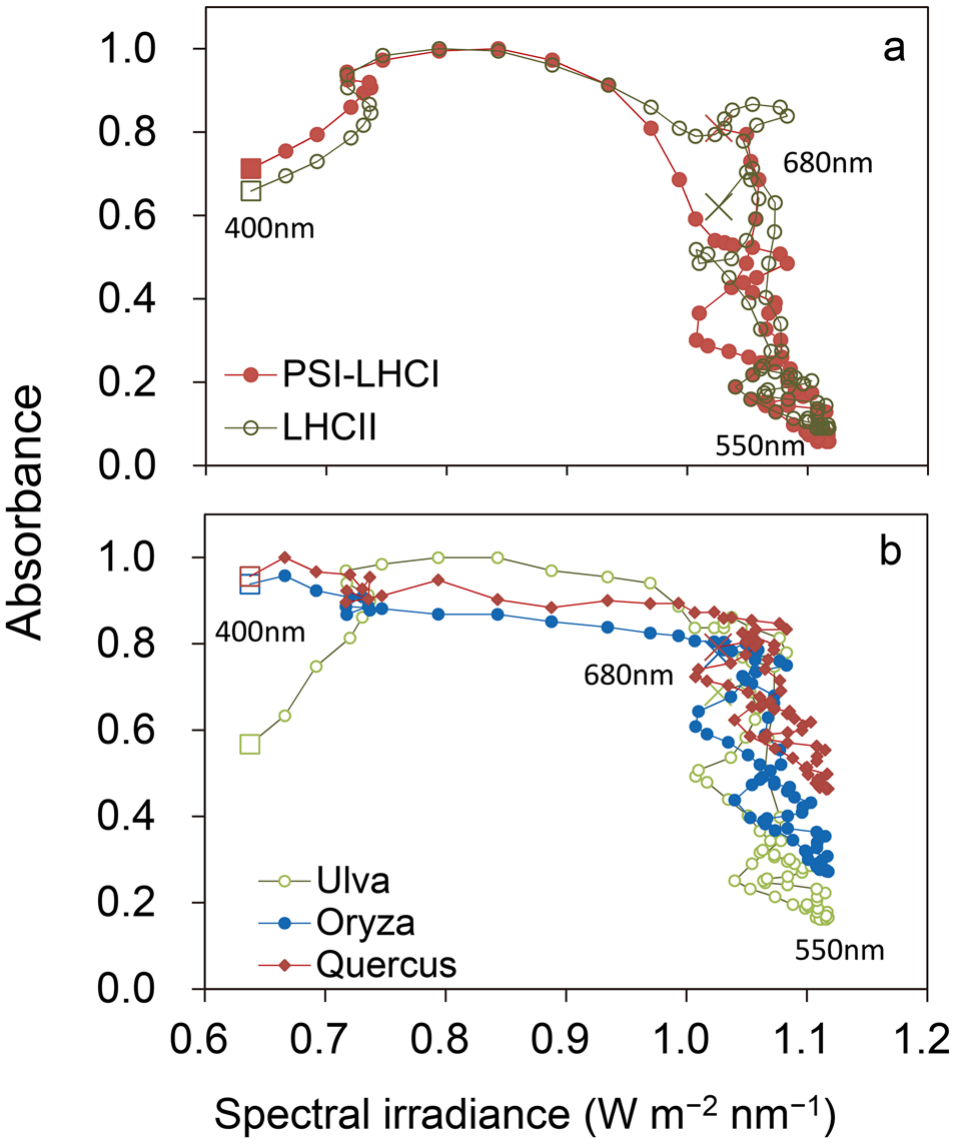
Relationships between spectral irradiance of direct solar PAR at noon and (**a**) spectral absorbance of purified LHCII trimer and PSI-LHCI and (**b**) spectral absorbance of an *Ulva* thallus and the leaves of *Oryza* and *Quercus* (Kume et al. 2016). The graphs are plotted with spectral absorbance on the *y-axis* and the spectral irradiance on the *x-axis* at 3.35-nm intervals in the 400- to 680-nm bandwidth. Points with consecutive wavelengths are connected with a line. The points with the shortest (400 nm) and longest wavelengths (680 nm) are indicated by a *square* and a *cross*, respectively

The waveband of the green region (500–570 nm) is identical to that of strong directional solar irradiance during midday hours under clear-skies (Fig. 1b). Kume et al. (2016) reported that the spectral absorbance of photosystems and intact leaves decreases linearly with the increased spectral irradiance of direct solar PAR at noon in the high spectral irradiance waveband (450–650 nm) (Fig. 2). The spectral absorbance of Chl *a* also has a strong negative correlation with the spectral irradiance (W m^−2^ nm^−1^) of global solar PAR at noon (R^2^ = 0.76) (Kume et al. 2016). These facts suggest that terrestrial green plants are fine-tuned to reduce excess energy absorption by photosynthetic pigments rather than to absorb PAR photons efficiently.

This contrasts with photosynthesis that occurs under water. Larkum (2006) pointed out that enhancement of green to blue light absorption would be advantageous for organisms living in deep water. At depths with low light intensities, organisms acclimate to the prevailing blue-green light by increasing the amount of accessory pigments, such as phycobiliproteins (Kirk 2011), which can absorb nearly all of the available PAR photons. However, terrestrial plants do not use phycobilisomes to capture green light, but developed blue and green photon-filtering pigments, such as carotenoids and anthocyanins (van den Berg et al. 2009). Nishio (2000) and Terashima et al. (2009) discussed this concept and addressed the possible course of its development.

In this mini-review, I demonstrate that the safe use of direct solar radiation is a key concept for understanding the light absorption of terrestrial plant leaves. Some basic concepts are introduced to explain the effects of the spectral absorbance of terrestrial plants using available data and simple models. First, the typical characteristics of incoming solar radiation are explained with regard to energy and photons. Second, the energy balances of leaves and chloroplasts are analyzed. Third, the effects of absorptance and absorption spectra of chloroplasts are discussed based on a comparison between green and “virtual” gray chloroplasts. Fourth, the effects of pigment concentrations on spectral absorptance are demonstrated. Based on these discussions, the mutualistic relationships among leaf anatomical development, chloroplast characteristics, and accessory pigments are considered in terms of the effective use of strong direct solar radiation in the terrestrial environment.

## Solar radiation spectra

Radiation has properties of both waves and particles and can be expressed in terms of energy flux (W m^−2^) or photon flux (mol m^−2^ s^−1^). The energy (***e***) of a photon flux can be calculated from its wavelength (λ, m), and for a given λ of a mole of photons:

1 $${{e}_{\lambda }}= N_{\rm A}hc/\lambda ,$$

where ***N***_**A**_ is Avogadro’s number (6.022 × 10^23^), *h* is Plank’s constant (6.63 × 10^−34^ J s), and *c* is the speed of light (3 × 10^8^ m s^−1^). According to this equation, shorter wavelength radiation has a higher energy content than longer wavelengths. Therefore, the solar radiation spectra can be described in terms of energy (irradiance) or photons, which results in different profiles (Fig. 1). For direct solar radiation on a sunny day, green light will be predominant for energy units (Fig. 1b) but red light (620–700 nm) will be so for photon units (Fig. 1c). As a result, the light-use efficiency differs depending on whether it is calculated based on energy or photon units. Energy-based radiation spectra are commonly used in meteorology, whereas photons are commonly used in photosynthetic studies because photosynthetic photochemical reactions are driven by photons. Thus, the photon flux density within PAR is commonly used in photosynthetic studies.

An incident solar beam is scattered by molecules or particles in the atmosphere and its directional and spectral properties are altered. We can conveniently define direct radiation as that which occurs from the radiation of the sun within a 5° angle and diffuse radiation as radiation that is not from the direction of the sun. Both irradiance and photon flux density spectra differ between direct and diffuse radiation in their magnitudes and profiles (Fig. 1b, c). Global radiation is the sum of direct radiation and diffuse radiation (Fig. 1a). The highest spectral irradiance of global radiation is observed in the 450–560-nm waveband at noon (Fig. 1a), but those of direct and diffuse radiation are in the 530–580-nm and 450–480-nm wavebands, respectively, at noon (Fig. 1b).

## Energy balance

The energy balance of a leaf is described based on the principle of the conservation of energy:

2 $${{\mathbf{R}}_{\mathbf{n}}}-\mathbf{C}-\varLambda \mathbf{E}=\mathbf{M}+\mathbf{S},$$

where **R**_**n**_ is the net radiation exchange, **C** is the net sensible heat loss, *Λ* **E** is the net latent heat loss, **M** is the net heat stored biochemical reactions of photosynthesis, and **S** is the net physical storage. Here, all these fluxes are expressed as per unit area of the leaf or projected area of organs (W m^−2^). For a thin leaf lamina, the flux into **S** is small and can be ignored. The rate of metabolic storage is dominated by photosynthesis. Typical maximum rates of net photosynthesis of 0.5–2.0 mg CO_2_ m^−2^ s^−1^ correspond to **M** values between 8 and 32 W m^−2^, which are usually less than 5% of **R**_**n**_ (Jones 2014). In addition, under high light conditions, **M**/**R**_**n**_ may decrease because of physiological and stomatal limitations. Therefore, **R**_**n**_ can be approximated by:

3 $$\mathbf{R}_\mathbf{n} =\mathbf{C} +{\varLambda} \mathbf{E},$$

where *Λ* is the latent heat of the vaporization of water (2.44 MJ kg^−1^ at 25 °C) and is the evaporation of water. **E** is driven by the vapor pressure deficit of the leaf surface (VPD_l_), which increases with increasing leaf temperature (*T*_leaf_). When *T*_leaf_ and air temperature (*T*_air_, surrounding temperature) are the same (i.e. **C** = 0), **E** increases linearly with **R**_**n**_. However, when **E** is zero, **C** increases with *T*_leaf_ and the difference between *T*_leaf_ and *T*_air_ will increase. **R**_**n**_ is also described as the difference between the total incoming radiation absorbed and the total longwave radiation emitted:

4 $$\begin{gathered}\mathbf{R_n} = \mathbf{I_s} ~\alpha _{\rm s} - \mathbf{L} \hfill \\ \;\quad \simeq \mathbf{I_s} ~\alpha _{\rm s} - 2\sigma \left( {T_{{{\text{leaf}}}}^{4} - T_{{{\text{air}}}}^{4} } \right), \hfill \\ \end{gathered}$$

where **I**_s_ is the radiation flux incident per unit area of the leaf surface (W m^−2^), α_s_ is the shortwave absorptance, **L** is the total longwave radiation emitted, ${\sigma }$ is the Stefan Boltzmann constant (5.67 × 10^−8^ W m^−2^ K^−4^). In the above equation, **L** can be estimated from the difference in the fourth power of *T*_leaf_ and *T*_air_.

## The absorption spectrum and radiation absorption of leaves

**Fig. 3:**
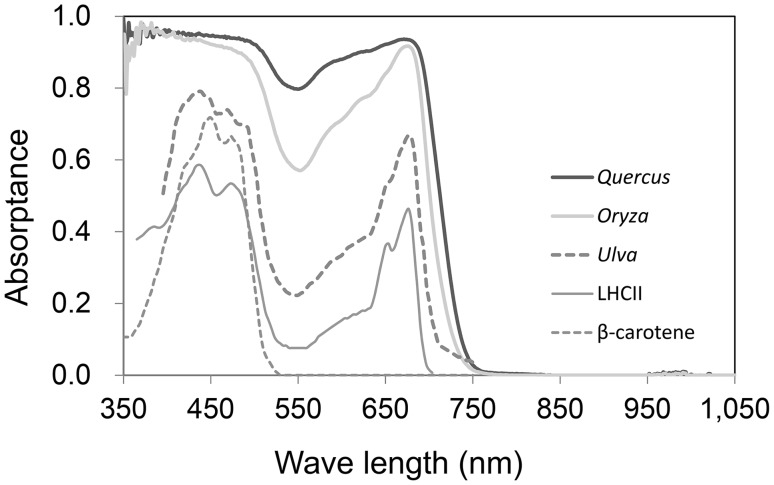
Absorptance spectra of the LHCII trimer (Hogewoning et al. 2012), β-carotene (Lichtenthaler 1987), a green alga thallus (*Ulva taeniata*) (Haxo and Blinks 1950), a grass leaf (*Oryza sativa*) and a tree leaf (*Quercus crispula*) (Noda et al. 2014). The absorptance of the LHCII trimer is adjusted to 0.3 for PAR and that of β-carotene is adjusted to 0.2 for PAR

Whether radiation is absorbed or not is dependent on the wavelength of the radiation and on the nature of the absorber. The absorption spectra (Fig. 3) are only important when considered within the spectra of incident radiation (Fig. 1). The maximum shortwave irradiance at midday is approximately 1,000 W m^−2^ in the summer over much of the Earth’s surface. However, plant leaves and their pigments do not absorb most photons that have wavelengths longer than 700 nm (Fig. 3; Kume et al. 2011). As a result, the absorbable solar radiation is less than 500 W m^−2^ (α_s_ ≤ 0.5). In terms of the total radiation balance of a leaf (**R**_**n**_), the thermal radiation flux (**L**) usually reduces the net radiation and **R**_**n**_ becomes approximately 80% of **I**_**s**_α_s_. In this case, the corresponding **E** value is approximately 9.0 mmol H_2_O m^−2^ s^−1^ (0.58 mm h^−1^); this is comparable to the maximum transpiration flux from closed forest canopies, which may be the densest absorber of PAR. The expected increase in leaf temperature is less than 10 °C (Okajima et al. 2012). If we assume the leaf is a black body (α_s_ = 1), these values will more than double. Therefore, the PAR-restricted absorption of leaves is effective at reducing transpiration and leaf temperature and serves as the fundamental means of reducing surplus energy absorption.

The total irradiance of PAR is approximately 0.45 of global solar radiation and is higher in summer (0.465) and lower in winter (0.420) in the mid-latitudinal areas of Japan (Akitsu et al. 2015). Plant leaves preferentially absorb red and blue wavebands, while the green region of incident light is absorbed less (Fig. 3). Approximately half of that unabsorbed radiation is reflected, leading to the green appearance of leaves. Therefore, such relationships may have some effects on leaf energy balance and photon absorption. To confirm the interactions between the spectral absorptance of leaves and the different classes of incident solar radiation (direct and diffuse radiation), the absorption of irradiance and photons in different leaf types were calculated for the mean solar spectral radiation at noon (11:30 am to 12:30 pm), based on spectral photon density (photons m^−2^ s^−1^ nm^−1^) and irradiance (W m^−2^ nm^−1^), for both direct and diffuse radiation (Table 1). Although each type of radiation has a different spectral distribution (Fig. 1), absorptance was almost the same between radiation types for *Oryza* (0.78) and *Quercus* (0.86) according to both photon- and energy-based calculations. *Ulva*, which is a green sea alga, showed a roughly 3% difference between direct and diffuse radiation. Therefore, the effects of spectral differences between direct and diffuse solar radiation are negligible for whole-leaf absorption properties and the absorption spectra of the intact leaves of terrestrial plants are nearly in unity (a gray body for PAR). The green color of leaves is conspicuous to the human eye, but insignificant for the energy balance of intact leaves.

**Table 1:** Irradiance- and photon-based description of the absorption of incident solar radiation (PAR) in various leaf types (*Ulva taeniata, Oryza sativa* and *Quercus crispula*) with different spectral absorptance values (Fig. 3) under different radiation classes (Fig. 1)

	Incident irradiance (W m^−2^)	*Ulva* (W m^−2^)	*Oryza* (W m^−2^)	*Quercus* (W m^−2^)
Global	400	196 (0.49)	311 (0.78)	346 (0.86)
Direct	281	135 (0.48)	217 (0.77)	242 (0.86)
Diffuse	119	61 (0.51)	94 (0.79)	104 (0.87)

	Incident photons (µmol m^−2^ s^−1^)	*Ulva* (µmol m^−2^ s^−1^)	*Oryza* (µmol m^−2^ s^−1^)	*Quercus* (µmol m^−2^ s^−1^)
Global	2000	951 (0.48)	1539 (0.77)	1717 (0.86)
Direct	1423	664 (0.47)	1089 (0.77)	1218 (0.86)
Diffuse	577	286 (0.50)	450 (0.78)	489 (0.86)

Values in parentheses are absorptance. Global radiation + diffuse radiation

## Energy absorption of a single chloroplast

The radiation absorption of a leaf arises as a consequence of absorption by chloroplasts because the absorptivity of other leaf cell organs is usually low or negligible (Hogewoning et al. 2012; Vogelmann and Gorton 2014). Therefore, increases in leaf temperature occur mainly because of the radiation energy absorbed by chloroplasts, which is transformed to thermal energy and transferred to surrounding cellular tissues. The light incident of a leaf, especially green light, is scattered and absorbed in the leaf (Brodersen and Vogelmann 2010; Terashima et al. 2009). To understand the initial process of light absorption in the leaf, we need to consider the absorption characteristics of a chloroplast.

**Fig. 4:**
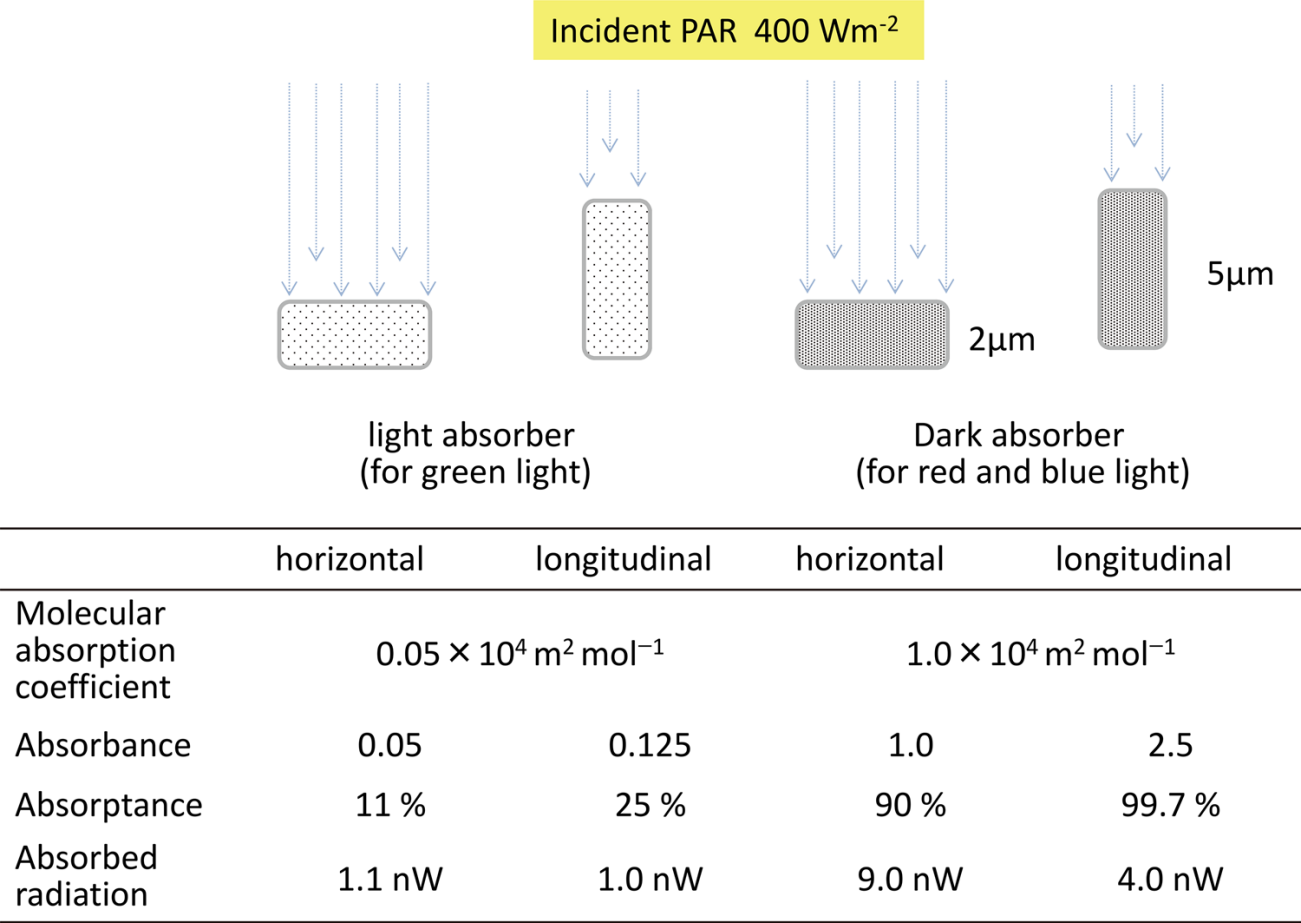
Model explaining the energy absorption of a chloroplast-like absorber with a different molecular absorption coefficient and position. The size of the absorber is 5 μm × 5 μm × 2 μm; the projected area of the short axis is 25 μm^2^, and the area of the long axis is 10 μm^2^. It is assumed that a 400 W m^−2^ of beam of incident radiation reaches the absorber

Here, we consider a typical chloroplast, as described by Terashima et al. (2009) and Terashima (2013). The assumptions for this simplified example are that the chloroplast is a cuboid sac containing a chlorophyll solution at a concentration of 50 mol m^−3^, with a size of 5 μm × 5 μm × 2 μm (Fig. 4). In this case, the projected area in the direction of the short axis is 25 μm^2^ and the area of the long axis is 10 μm^2^. To mimic the case of strong blue and red light absorption, we estimated that the absorption coefficient of the pigment in the solution is 1.0 × 10^4^ m^2^ mol^−1^. The absorbance (*A*) of the short axis direction becomes 1, and 90% of incident light is absorbed. The *A* of the long axis direction is 2.5, and 99.7% of the light is absorbed. If we assume a 400 W m^−2^ incident beam of radiation on the chloroplast, then the amount of absorbed radiation per chloroplast can be calculated by multiplying its projected area. When the beam is incoming from the short axis direction or from the long axis direction, then the total absorbed radiation is 9.0 or 4.0 nW, respectively (Fig. 4). This result indicates that the effects of chloroplast angle on the light beam can more than double the radiation absorption.

Next, we assumed that the absorption coefficient was 0.05 × 10^4^ m^2^ mol^−1^ to mimic the weak absorption of green light. In this case, the *A* of the short axis direction becomes 0.05 and 11% of the incident light is absorbed; further, the *A* of the long axis direction is 0.125 and 25% of the light is absorbed. When the 400 W m^−2^ beam is incoming from the short axis direction or from the long axis direction, the total absorbed radiation is 1.1 or 1.0 nW, respectively (Fig. 4). That is, the weak absorptance of green light (the low molecular absorption coefficient) means constant low absorption of light regardless of the chloroplast position in the cell; this considerably impacts suppression of the absorption of strong direct radiation.

## Energy absorption of a series of chloroplasts

**Fig. 5:**
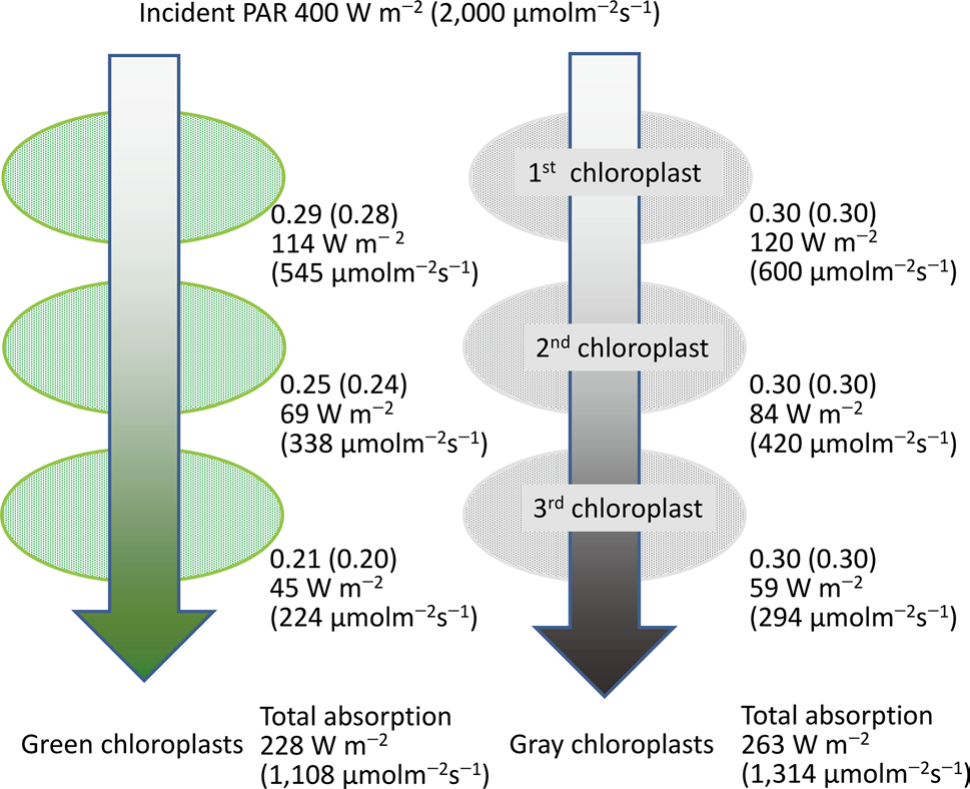
Model explaining the energy balance of a series of three chloroplasts with the same absorptance. *Left*: cuvettes containing a green pigment solution with the same spectral absorptance as that of LHCII. *Right*: cuvettes containing a *gray* solution. Both *colored cuvettes* have 0.3 of PAR absorptance (see Fig. 6a). Incident beam PAR radiation has the same spectral properties as the global solar radiation at noon (Fig. 1) and is 400 W m^−2^ of irradiance or 2,000 μmol m^−2^ s^−1^ of PFD (values in *parentheses*)

Many chloroplasts exist in mesophyll cells and their layers reflect, absorb, or transmit incident radiation. A series of absorption processes occur in many absorbers in a leaf. These processes can be considered using a simple model (Fig. 5). We assumed that the gray chloroplasts, with an average PAR absorptance (α_PAR_) of 0.3 and an incident solar radiation of 400 W m^−2^, have the same spectral direct solar radiation profiles (Fig. 1). The first gray chloroplast absorbs 120 W m^−2^ of radiation.

**Fig. 6:**
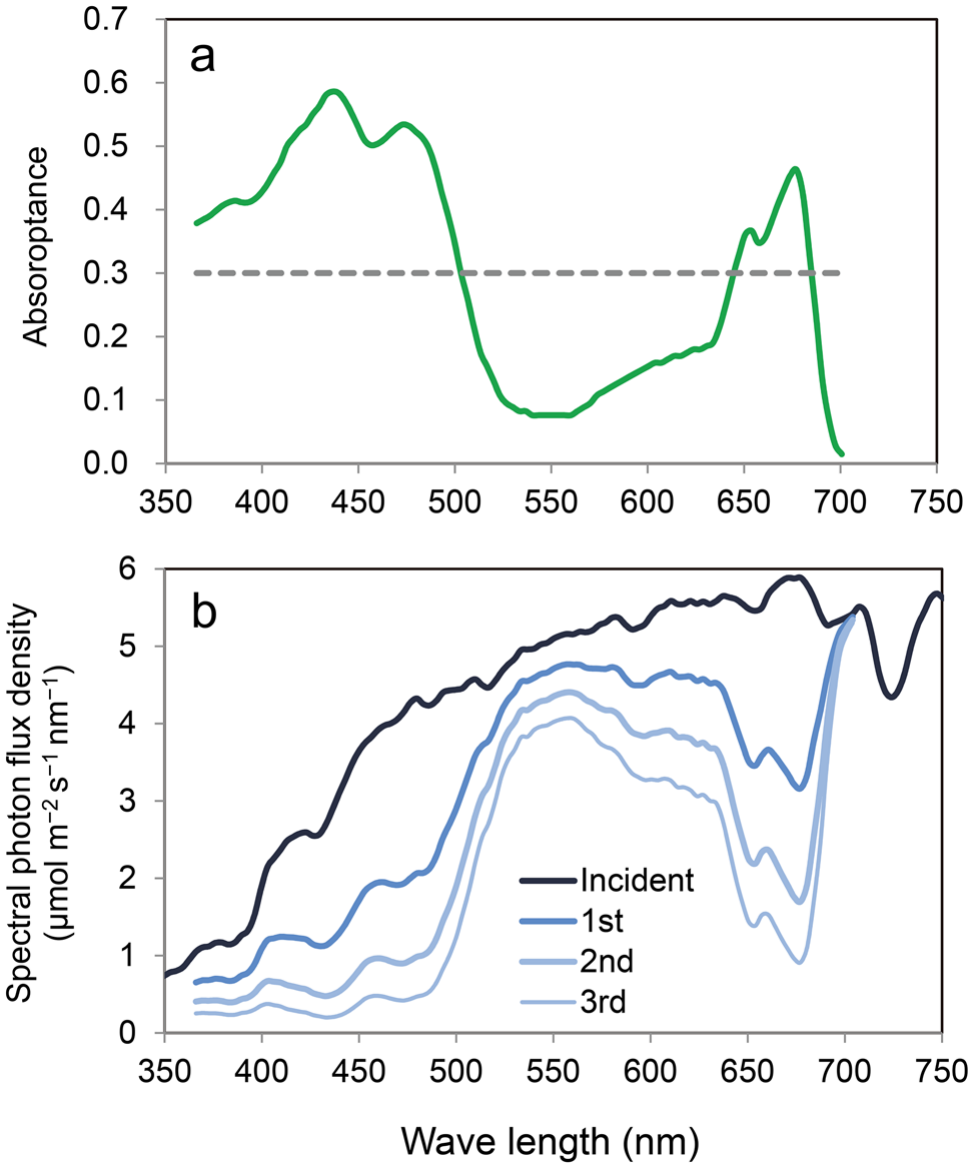
**a** Spectral absorptance of the gray body (*dashed line*) and simplified green chloroplast (*solid line*). In both cases, the mean PAR absorptance is 0.3. **b** Changes in spectral irradiance by the absorption of three green chloroplasts. The ratio of green light components exponentially increased at each absorption step

Next, to mimic green chloroplasts, the absorbance profile of the light harvesting complex II (LHCII) trimer (Fig. 3) was applied and adjusted to the mean α_PAR_ of 0.3 (Fig. 6a). LHCII is the major light-harvesting complex of plants and the most abundant membrane protein. The absorption spectrum of the LHCII trimer is highly correlated with that of an *Ulva* thallus (R^2^ = 0.98), which has a simple morphological structure with two thin, flat layers of cells; thus, it may represent the average chloroplast absorption spectrum. In this case, the first green chloroplast absorbs 114 W m^−2^ of radiation (Fig. 5). For the absorption process of a series of three gray chloroplasts, each chloroplast absorbs 0.3 of incident radiation regardless of the wavelength (Fig. 6a). The third chloroplast absorbs 59 W m^−2^ of radiation and the total absorbed radiation by the three chloroplasts is 263 W m^−2^ (Fig. 5).

However, the absorption process of a series of three green chloroplasts is slightly different because the ratio of green light photons increases in each chloroplast (Fig. 6b). As a result, the absorptance of the second and the third chloroplast decreases to 0.23 and 0.20, respectively (Fig. 5). The third chloroplast absorbs 45 W m^−2^ of radiation, which is 76% that of the third gray chloroplast. The total absorbed radiation by the three green chloroplasts is 228 W m^−2^, which is 87% of that absorbed by the gray chloroplasts. Using photon-based calculations, the results are similar, with the green chloroplasts absorbing 1108 μmol m^−2^ s^−1^, which is 84% of the total photons absorbed by the gray chloroplasts (Fig. 5). Thus, the absorption spectra of chloroplasts have important effects on the reduction in radiation absorption during the initial process of leaf photon absorption. Notably, a strong scattering and chandelier effect (Terashima et al. 2016) may occur in real mesophyll tissues and the analyses of transmission and reflection process must be evaluated if the whole light absorption process within a leaf is considered (Terashima et al. 2009; Xiao et al. 2016).

## Energy balance of chloroplasts and the absorptance gradient effect

To maintain the energy balance of chloroplasts in a cell, the absorbed radiation energy increases the chloroplast’s temperature (making it a heat generator) and the thermal energy is then dispersed from the surface of mesophyll cells (the heatsink) in the form of latent heat through transpiration from the cell surface. In this case, the higher cell surface area per chloroplast might be advantageous in terms of the chloroplast’s cooling efficiency. Nobel (1977) reported that the mesophyll cell wall area per unit leaf area ranged from 10 for leaves in the shade to 35 for leaves in the sun. Oguchi et al. (2005) showed that the inherent mesophyll surface area development of different species determines their light acclimation potential. When we assume a leaf with a 3 mmol m^−2^ s^−1^ transpiration rate and 15 m^2^ m^−2^ of chloroplasts facing the intercellular space per unit leaf area, the transpiration rate per mesophyll surface area becomes 0.2 mmol m^−2^ s^−1^; this corresponds to 8.8 W m^−2^ of latent heat flux. If we assume the area facing the intercellular space area to be 25 μm^2^, it is comparable to 0.22 nW per chloroplast. Usually, chloroplasts cover nearly all of the mesophyll cells’ surface area (Oguchi et al. 2005). Thus, the above value may be the maximum capacity of the latent heat transport for a chloroplast. However, this value is relatively small compared with the magnitude of the incident solar radiation (Fig. 4). In addition, stomatal closure is a sensitive response to water deficits and the leaf-air temperature differential can be used as a measure of the degree of water stress under high light conditions (Idso 1981). This means that under water-stressed conditions, the effects of sensible heat loss (***Λ*** **E**) and photosynthetic metabolism (**S**) become negligible, and all of the absorbed radiation must be dissipated as heat (rising protoplast temperature). Under such conditions, when we assume the first gray chloroplast absorbs 120 W m^−2^ of radiation (Fig. 5), the temperature of the first chloroplast may rise by 10 °C to maintain the system’s energy balance. For green chloroplasts, the result is almost the same. However, the third gray chloroplast absorbs 59 W m^−2^ and the green chloroplast absorbs 45 W m^−2^, resulting in 5 and 4 °C increases, respectively. This simple calculation suggests that if the absorptance of chloroplasts was the same within a leaf, then there would be significant temperature differences generated between the upper and lower sides. The chloroplasts that face the irradiated surfaces would suffer strong heat stress or be in a state of having excess energy. Notably, thermal conduction between organelles is quite high, and the temperature becomes uniformly distributed within a short period. In addition, although the cooling effects of latent heat loss are limited, they may influence internal leaf temperature equilibration because evaporation will be accelerated where the cell surface temperature is relatively high in the mesophyll space.

As we discussed in the preceding section, the effects of the absorber’s absorptance are important in alleviating a convergence of radiative absorption. In fact, a clear gradient in chloroplast properties from sun- to shade-type chloroplasts exists within leaves (Nishio et al. 1993; Terashima and Inoue 1984, 1985). The chlorophyll concentration of the inner leaf is approximately two to three times higher than that near the adaxial surface of the leaf. When tissues from the same leaves are compared, the apparent absorption coefficients are greater in the spongy tissue than in the palisade tissue. Terashima et al. (2016) highlighted that the chlorophyll profile shows a clear sub-surface optimum and that the chloroplasts in the first cell layer are pale green while those in the second layer are a much deeper green.

**Fig. 7:**
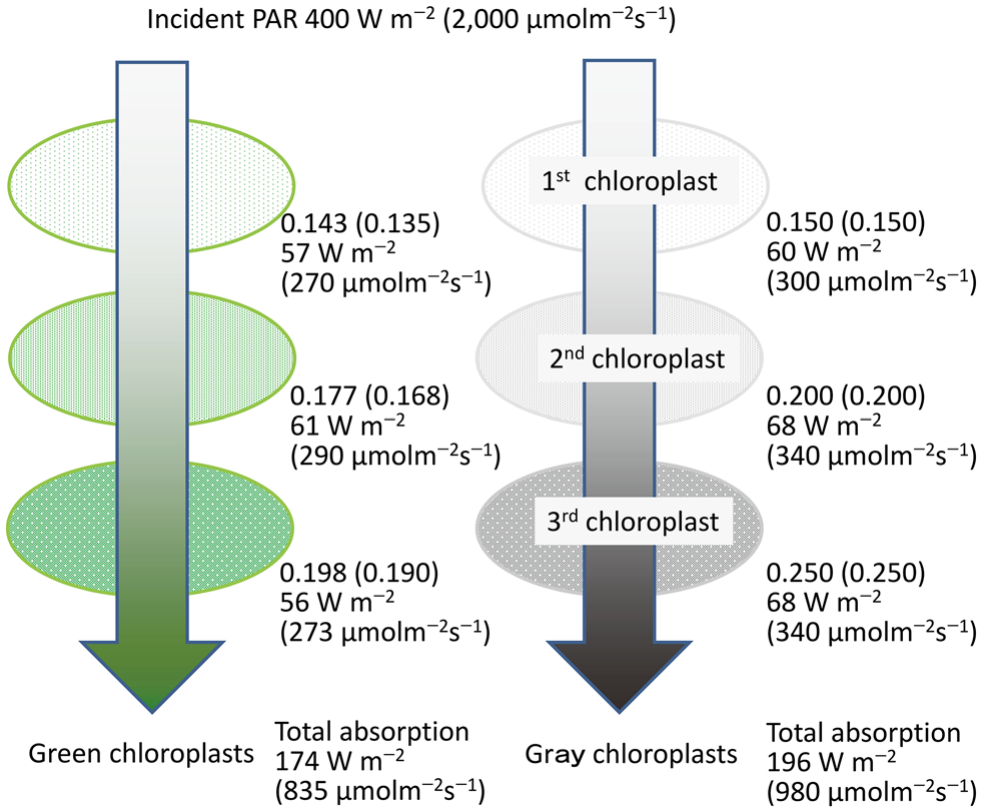
Model explaining the energy balance of a series of three chloroplasts with absorptance gradient. *Left*: cuvettes containing a green pigment solution with the same spectral absorptance as LHCII. *Right*: cuvettes containing a *gray* solution. The *colored cuvettes* have 0.15, 0.20, and 0.25 PAR absorptance from *top* to *bottom*, respectively. The incident beam PAR radiation has the same spectral properties as the global solar radiation at noon (Fig. 1), with 400 W m^−2^ of irradiance or 2000 μmol m^−2^ s^−1^ of PFD (values in *parenthesis* are for PFD)

To mimic such situations, three chloroplasts with an absorptance gradient of 0.15, 0.20, and, 0.25 were assumed (Fig. 7). In this case, the energy absorption of each chloroplast is fairly balanced and all of the chloroplasts absorb approximately the same amount of radiation, regardless of their order. These chloroplasts absorb approximately 60 W m^−2^ of energy and a 4 °C increase is expected. The total radiation absorbed by the three gray chloroplasts is 196 W m^−2^, while green chloroplasts absorb 174 W m^−2^, approximately 10% less energy absorption. The results of photon-based calculations are similar to those of energy-based calculations for this phenomenon, but the effects of the spectral absorptance on photon absorption suppression are greater. The total photons absorbed by the three gray chloroplasts are 980 μmol m^−2^ s^−1^, but the green chloroplasts absorb 835 μmol m^−2^ s^−1^, which is approximately 14% less (Fig. 7). Thus, the effect of the lower absorptance of chloroplasts reduces photon absorption in the initial absorption process and the combined effects of the mean absorptance gradient and the spectral absorptance of chloroplasts are notably large.

## Anatomical characteristics

**Fig. 8:**
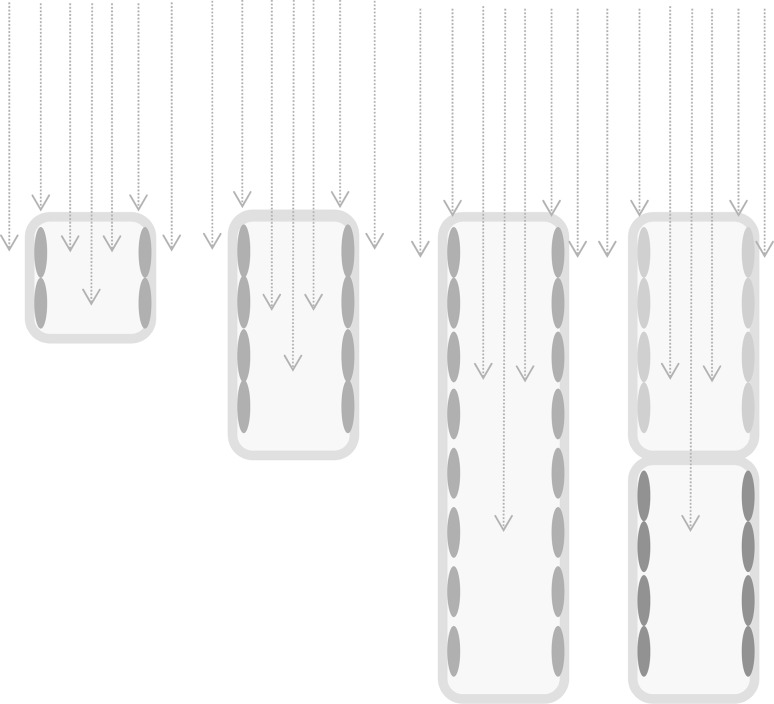
Model explaining the effects of the aspect ratio of palisade cells (cell length per column diameter) on the absorption areas of chloroplasts. The effective absorption area increases with the aspect ratio. Therefore, the absorption of photons of direct radiation per chloroplast is nearly inversely proportional to the aspect ratio. The stacked palisade cells with the absorptance gradient in chloroplasts (the *right* end) can absorb photons more efficiently

The chlorophyll concentration gradient exerts considerable effects on the radiation absorption process of chloroplasts, and inner chloroplasts may absorb more radiation than surface chloroplasts (Oguchi et al. 2011). To reduce the amount of the initial absorption of direct solar radiation, the thicker structure of the leaf is effective at dispersing the absorbable radiation per chloroplast because palisade cells can transmit light deeper into the leaf (Fig. 8; Gorton et al. 2010; Smith et al. 1997). The well-developed palisade layer in a leaf consists of several tiers of columnar cells with well-developed vacuoles and allows direct light to penetrate into the lower layer (Brodersen and Vogelmann 2010; Vogelmann 1993). The incident photon flux on protoplasts is distributed along the vertical palisade cell surface. Such anatomical properties decrease the incident photons per total palisade cell surface area and decrease photon density per chloroplast surface area (Fig. 8). The ratio of the cell length to the column diameter is an important factor that helps determine the dilution of incident direct light. That is, a higher ratio (or more palisade layers) has a greater ability to balance light distribution within cells (Vogelmann and Martin 1993). This corresponds well with the observation that leaves in the sun tend to have more highly developed palisade tissue, with longer and thinner cells, than leaves in the shade (Hanba et al. 2002). Both sieve and detour effects (Terashima et al. 2009; Vogelmann and Martin 1993) may help to spread photons to a larger number of chloroplasts.

In many cases, the regulatory mechanisms of optical absorption in the photochemical system and the equalizing mechanisms of radiant energy absorption among chloroplasts perform similar functions. CO_2_ supply to chloroplast restricts photosynthesis and the balance between the radiant energy inflow and the CO_2_ flux in a chloroplast under high light conditions requires a reduction in PAR photon absorption per chloroplast. The reduction also balances the thermal distribution. “Dark” chloroplasts with high absorptance tend to absorb incident radiation strongly, concentrating energy in a small space. Therefore, lower-absorptance chloroplasts are required in higher light environments.

In shade environments, leaf darkening can improve light absorptance. Shade-grown leaves increase their light-harvesting pigment concentration and decrease the ratio of the thickness of the palisade layer to that of the spongy parenchyma layer (Givnish 1988). The development of grana, multiple layers of thylakoids, is effective for concentrating light-harvesting pigment density (Nishio 2000) and makes chloroplasts darker. The total absorptance of a leaf does not differ greatly between leaves in the sun and those in the shade, but the total absorption area of chloroplasts in shade-grown leaves is quite small (Evans and Poorter 2001). Therefore, PAR photon absorption per chloroplast may increase. Leaves grown under high light conditions with multiple palisade layers showed better utilization of direct than diffuse light, while shade-grown leaf structure showed no preference for direct or diffuse light at any irradiance level (Brodersen et al. 2008).

**Fig. 9:**
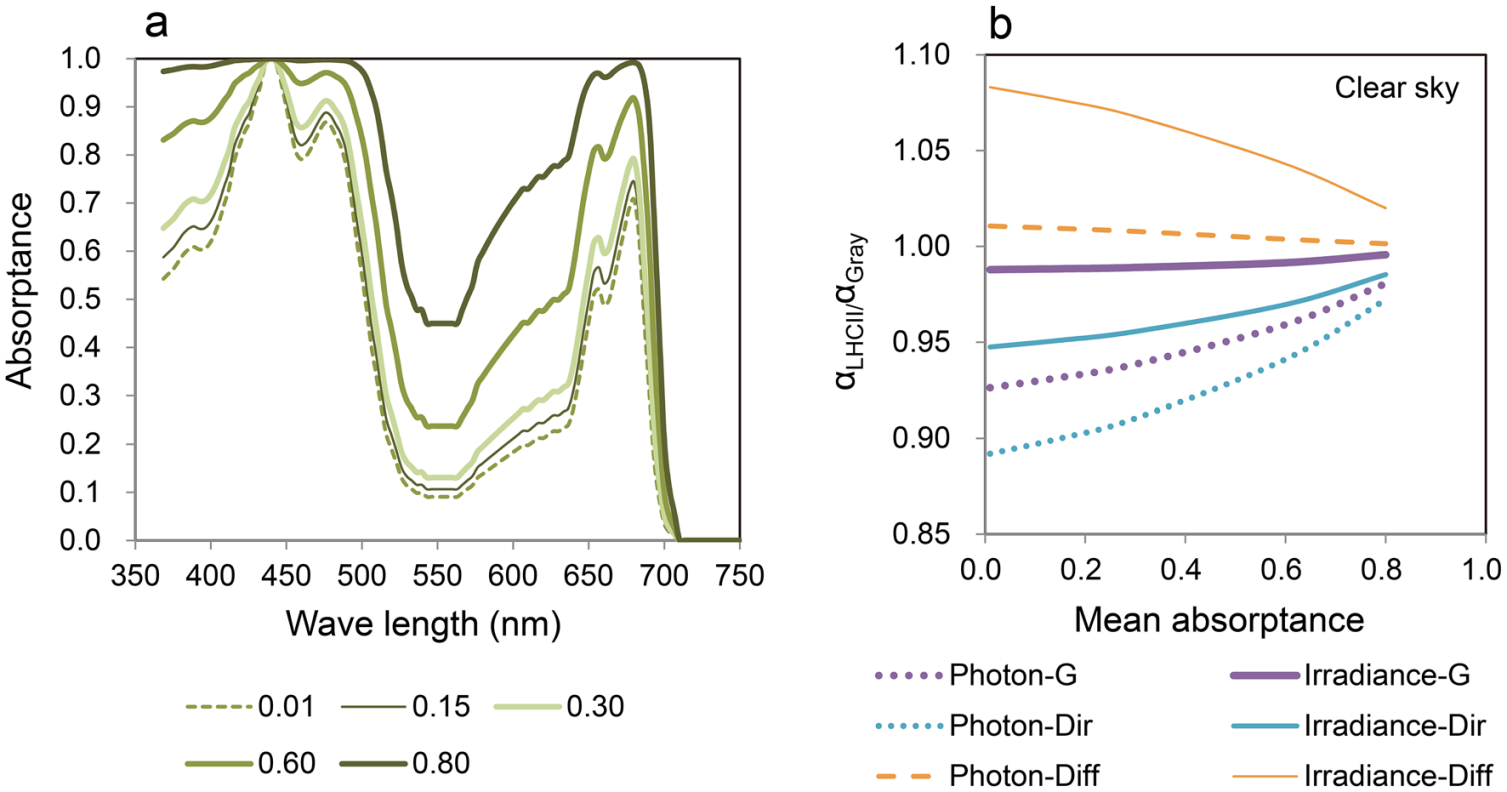
Effects of pigment concentration on changes in the profile of absorptance spectra. **a** Profiles of the spectral absorptance of LHCII with different mean PAR absorptance values (mean PAR absorptance). The spectra were normalized to the maxima of the Soret bands. In real leaves, multiple absorptions within leaf tissues decrease the absorption depression in the green region (detour effect). **b** The ratio of the absorptance of LHCII (α_LHCII_) to that of the gray absorber with the same absorptance (α_Gray_) under different radiation classes (global, direct and diffuse; solar irradiance and photons; see Fig. 1). The suffix “–G” indicates global radiation, “–Dir” indicates direct radiation and “–Diff” indicates diffuse radiation

The regulatory effect of absorptance by the absorption spectrum is closely related to pigment concentration (Figs. 4, 9). Leaf to canopy-level optimization of light-use efficiency may be possible through different strategies of relatively low-absorptance leaves, such as those of graminoid species that do not have well-developed palisade tissues. Terashima et al. (2016) pointed out that the silica-rich light diffusive leaves of *Oryza sativa* do not show obvious transmittance changes and there are virtually no spaces for chloroplasts to move around in the cells of the mesophyll (Sage and Sage 2009). This could be related to the lower absorptance of *Oryza* leaves (Fig. 1; Table 1).

## Absorption spectrum and absorptance of photosystems

Absorptance is an important characteristic that determines the radiation energy balance of absorbers, and the concentrations of photosynthetic pigment molecules largely determine the absorptance of chloroplasts and leaves. When the pigment concentration is very low, the profile of spectral absorptance is nearly equal to spectral absorbance, but when the concentration is very high, the spectral absorptance would reach unity and approach that of a black body. As seen in Fig. 9a, the profile of the absorption spectra of LHCII is nearly the same as the profile of absorptance spectra when the concentration is very low (PAR absorptance = 0.01). However, when the concentration is high (PAR absorptance = 0.80), the profile of the absorptance spectra becomes flat especially in the short waveband range (400–500 nm).

To evaluate the effect of differences in the absorption spectra, the absorption of LHCII (α_LHCII_) is compared with that of a gray absorber of the same PAR absorptance (α_Gray_; see Fig. 6a). The ratio of α_LHCII_/α_Gray_ for a certain PAR absorptance is calculated for different types of solar radiation spectra (Fig. 9b). When α_LHCII_/α_Gray_ is below unity, the green chloroplast absorbs less radiation than the gray chloroplast with the same PAR absorptance (e.g. Figs. 5, 7). In Fig. 9b, the *y*-axis indicates α_LHCII_/α_Gray_, and these converge into unity (akin to a black body) with PAR absorptance (concentrations). When the mean absorptance is very low (0.01), the α_LHCII/_α_Gray_ ratio of the photons from direct solar radiation is 10% lower (Fig. 9b, Photon-Dir) and that of global radiation is approximately 7% lower (Fig. 9b, Photon-G). α_LHCII/_α_Gray_ of the irradiance from direct solar radiation is also 5% lower (Fig. 9b, Irradiance-Dir), but that of diffuse radiation is approximately 8% higher (Fig. 9b, Irradiance-Diff). This is because each radiation spectra has a different peak wavelength (Fig. 1), and the spectra of α_LHCII_ adjust to avoid the spectra of directional beam radiation, preferring that of diffuse radiation (Kume et al. 2016).

Photon absorption from global and direct solar radiation near noon is considerably reduced because of the spectral absorption characteristics of LHCII (Fig. 9b, Photon-G and Photon-Dir), regardless of the cloud conditions (data not shown). However, irradiance absorption from global solar radiation is not greatly reduced because diffuse irradiance is strongly absorbed (Fig. 9b, Irradiance-G and Irradiance-Diff), though the absorption reduction in irradiance is observed from direct solar radiation (Fig. 9b, Irradiance-Dir). Therefore, the total spectral effect on absorption becomes small for global radiation energy. Brodersen and Vogelmann (2010) showed that diffuse light tended to be absorbed more in shallower tissue parts. The absorption spectra of diffuse solar radiation may also facilitate light absorption in shallow leaf tissue.

Such spectral effects on light absorption are obvious until mean absorptance reaches 20%, which is the approximate absorptance of a chloroplast (Fig. 9b). However, there is almost no effect at a mean absorptance of approximately 80%, which is the absorptance of a whole leaf (also see Table 1). Spectral effects on the light absorption are effective for chloroplasts, but not individual leaves.

## Filtering effects of accessory pigments

Wavelength dependence on photon yield for CO_2_ fixation is nearly equal under ideal conditions if there is no absorption by carotenoids and nonphotosynthetic pigments (Hogewoning et al. 2012). Although blue light has higher energy per photon than red light, the photosynthetic efficiency per absorbed photon of blue light is equal to that of red light. However, excited states resulting from the absorption of blue photons are degraded within subpicoseconds to the level of red ones before they are used (Björn et al. 2009). In this process, energy is exchanged intramolecularly as heat. Therefore, even if the photon yield of CO_2_ fixation does not differ, more heat is generated by the use of blue photons than red photons.

**Fig. 10:**
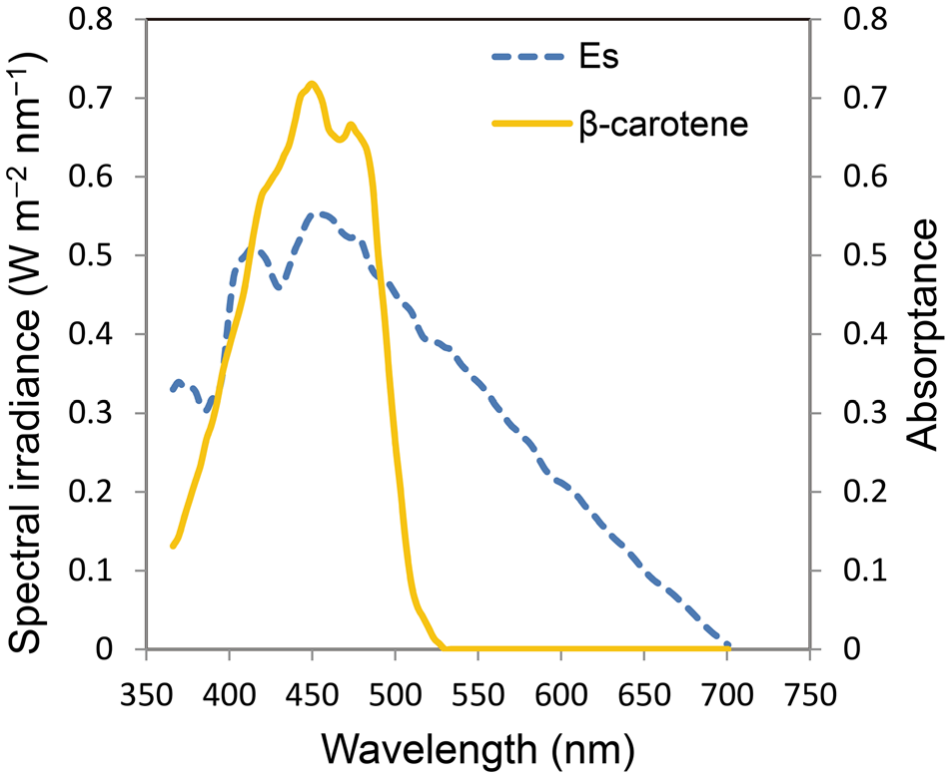
Absorption spectra of β-carotene with 0.2 of PAR absorptance (no units) and energy spectra of surplus energy for photosynthesis (**Es**) (W m^−2^ nm^−1^). **Es** was calculated from Eq. (5) (see also Fig. 1a)

Here, we can define the surplus energy (**Es**, W m^−2^ nm^−1^) on the basis of the energy of 700 nm photons,

5 $${\mathbf{Es}}\, = \, P_{{(\lambda )}} \times {\text{ }}\left( {e_{\lambda } - e_{{700}} } \right),$$

where *P*_(λ)_ is the incident spectral photon flux density at (μmol m^−2^ s^−1^ nm^−1^), e_λ_ is the photon energy at λ (J), and e_700_ is the photon energy at 700 nm (2.84 × 10^−19^ J). The **Es** of global radiation has a peak at approximately 450 nm (Figs. 1a, 10). Thus, the incidence radiation energy that is potentially exchanged as heat has a peak near 450 nm. The total amount of **Es** is approximately 87 W m^−2^ per 400 W m^−2^ of total PAR irradiance (22%). Therefore, to reduce the absorption of **Es** effectively, it is important for absorption in the short waveband (400–500 nm) to be restricted. However, the leaf absorptance of this region is quite high (Fig. 3) because of the existence of carotenoids as well as the high absorbance peak of chlorophylls.

Carotenoids act as light absorbers in light-harvesting complexes, but the energy transfer efficiency of β-carotene is about 35% (de Weerd et al. 2003). Additionally, carotenoids perform an essential photoprotective role within the chloroplast (Johnson et al. 2011; Young 1991) and the violaxanthin-cycle carotenoids effectively absorb blue light and do not transfer absorbed light to chlorophyll (Nichelmann et al. 2016). These leaf pigments show significant absorption of photons that have wavelengths shorter than 520 nm, as seen in the leaf absorptance spectra of albino cucumber leaves (Hogewoning et al. 2012). Thus, carotenoids absorb blue light and decrease its ability to drive photosynthesis by effectively preventing the inflow of blue light photons into the photochemical system. The importance of carotenoids in producing a “screening” effect to reduce blue light has been suggested (Hogewoning et al. 2012; Nishio 2000; Terashima et al. 2009). These mechanisms may not prevent heat absorption itself but reduce blue light absorption by chlorophylls.

If we assume that the 400 W m^−2^ of incident global PAR is absorbed by β-carotene with a 0.2 average absorptance for the PAR waveband (Fig. 10), then approximately 20% (82 W m^−2^) of global PAR will be absorbed. The PAR by absorbed by β-carotene has a larger **Es** ratio (Eq. 5); thus, it would contain 33% (29 W m^−2^) of total **Es**. Because the total carotenoid content in leaf tissues and chloroplasts could be greater than in this example, effective screening of **Es** inflows should occur in leaves. Chlorophyll pigments have high absorbance peaks at wavelengths around 400–500-nm, where the ratio of **Es** is high and the spectral absorbance of β-carotene is effective for eliminating photons that produce high **Es** (Kume et al. 2016).

Many “biological pigments”, such as DNA, and many proteins, hemes and porphyrins have their intrinsic absorption peaks in the short waveband region from ultraviolet to blue light. Reducing the absorption of blue light and the excitation of manganese is important for reducing photoinhibition (Hakala et al. 2005; Oguchi et al. 2011). To reduce the photon absorption of important microstructures in organelles, incident blue and UV photons must be absorbed by other defense pigments. For example, the absorbance spectrum of β-carotene is similar to the ideal band-pass filter, which absorbs purple to blue light without attenuation in yellow to red light (Kume et al. 2016). As a result, the ratio of yellow to red photons increases and **Es** inflow into photosystems decreases. Although there are many types of pigments, that absorb shorter wavelengths than carotenoids do, there are few types of pigments that absorb longer wavelengths. Even red anthocyanidin, which can absorb longer wavebands, passes mainly through the red photons (>600 nm).

Anthocyanins, which are observed in red leaves, mainly absorb green photons at 500–600-nm; this waveband corresponds with the peak of incident solar radiation irradiance. Therefore, leaves can increase their temperature rather than increase their protection against excess light for photosynthesis (Karageorgou and Manetas 2006; van den Berg et al. 2009). Kyparissis et al. (2007) showed a significantly lower level of palisade tissue development in the red leaves of *Prunus cerasifera* compared with that in the green leaves. They suggested that green light attenuation by anthocyanins may impose a limitation on leaf thickness. Further study is needed to clarify the ecophysiological roles of nonphotosynthetic pigments.

## Chloroplast displacement

A chloroplast can control red and blue photon absorption through its displacement and deformation. Furthermore, chloroplast movements are very important under high light conditions. Tholen et al. (2008) concluded that the avoidance of photoinhibition is the first priority for chloroplast movements rather than facilitating CO_2_ diffusion. *Arabidopsis thaliana* mutants that lack movement of their chloroplasts are seriously photoinhibited (Kasahara et al. 2002). Under experimental conditions, chloroplast movements cause an approximately 10% difference in the light transmittance of a leaf (Gabryś and Walczak 1980; Inoue and Shibata 1974; Trojan and; Gabryś 1996). However, the magnitude of this difference is attenuated under field conditions (Williams et al. 2003). In a natural environment, the frequency distribution of incident radiation is quite skewed and the duration of the high radiation period is short and intermittent (Miyashita et al. 2012). To guard against these unpredictable events, it is reasonable to have the lowest spectral absorption possible in the range of the highest direct solar radiation waveband.

Chloroplast movements in response to light are relatively ineffective under green light for energy balance and photon absorption but they are quite effective for blue and red light absorption (Fig. 4). This is corroborated by the fact that only blue light induces the directional movement of chloroplasts in the mesophylls of terrestrial angiosperms (Banaś et al. 2012). This phenomenon also helps to reduce light harvesting and acts as a form of protection against excess energy under high light conditions (Park et al. 1996; Sztatelman et al. 2010). Notably, when plants are grown under strong sunlight, their leaves may have sun-type chloroplasts, which have a low chlorophyll concentration and undeveloped thylakoids (Terashima et al. 1986). In this case, the effects of chloroplast displacement are expected to be diminished because of low absorptance. Higa and Wada (2016) observed that chloroplast avoidance movements do not occur in plants grown under strong sunlight.

## Conclusion

The optical design of plant leaves manages various complex factors, such as CO_2_ absorption, evasion of high light conditions, low light absorption, decreases in transpiration, and water-use efficiency, through anatomical leaf structures and chloroplast characteristics as well as pigment distribution and concentration. The main methods by which different levels of leaf components regulate energy are summarized in Table 2. Because incident solar radiation is too strong to be utilized safely for photosynthesis with the current terrestrial CO_2_ concentration in the atmosphere, the illuminated surface areas of chloroplasts in a leaf are enlarged and incident direct solar radiation is dispersed. Although leaf anatomical structures interact with the characteristics of the photochemical system, the absorption spectrum is the most fundamental way that photon absorption is controlled. Low absorption of infrared light contributes greatly to the lowering of leaf temperatures and hence to improving water-use efficiency in photosynthesis. The gradation of chlorophyll concentration in chloroplasts is important for control of the absorption of incident solar radiation within a leaf. The spectral characteristics of absorbers are important factors for the energy regulation of chloroplasts and smaller-scale energy processes. Chloroplasts have low absorptance spectra (except those of blue and red light), and blue photons, which contain much **Es**, are absorbed by accessory pigments such as carotenoids. Preventing excess energy absorption in photosystems is a primal survival strategy in terrestrial environments, where photon flux density can fluctuate by several orders of magnitude. Although these processes are tightly connected with the characteristics of terrestrial solar radiation, there are limited available data regarding precise spectral solar radiation for botanical research, and this topic has not been well covered in the literature. Careful consideration of the terrestrial radiation environment and photon absorption processes is important for understanding the evolution of embryophytes.

**Table 2:** Regulatory modes of energy balance within a leaf

	Leaf	Chloroplast	Photosystems and pigments
Latent heat	✓	△	×
Orientation and shape	✓	✓–△ (displacement)	×
Palisade tissue development	✓	✓	×
Absorption spectra (IR)	✓	✓	✓
Absorption spectra (PAR)	×	✓	✓
Absorptance	✓	✓	×–△
Pigment filtering by carotenoids	×	△	✓

‘Latent heat’ refers to the cooling effects of evaporation from the cell surfaces. ‘Orientation and shape’ are relative to the radiant beam. Chloroplasts can select suitable positions within palisade cells. Palisade tissue development mainly reflects the ratio of the cell length to the column diameter and the numbers of cell layers. ‘Absorption spectra (IR)’ refers to the effects of spectra with wavelengths longer than 700 nm on the energy absorption. ‘Absorption spectra (PAR)’ refers to the effects of spectra with wavelengths shorter than 700 nm. These effects do not include those of absorptance by absorbers or the concentration of pigments. ‘Absorptance’ refers to the effects of absorptance of absorbers or concentration of pigments. ‘Pigment filtering by carotenoids’ refers to the effects of blue and purple light absorption by carotenoids. As a result, chlorophylls can avoid shorter wave-length photons with high **Es**. This avoidance does not reduce total photon absorption but reduces the electron energy inflow into the photosystems
