# Correction to: Importance of the green color, absorption gradient, and spectral absorption of chloroplasts for the radiative energy balance of leaves

**DOI:** 10.1007/s10265-018-1014-0

**Published:** 2018-02-21

**Authors:** Atsushi Kume

**Affiliations:** Faculty of Agriculture, Kyushu University, 6-10-1 Hakozaki, Higashi-ku, Fukuoka 812-8581 Japan

## Correction to: J Plant Res (2017) 130:501–514 10.1007/s10265-017-0910-z

The article “Importance of the green color, absorption gradient, and spectral absorption of chloroplasts for the radiative energy balance of leaves”, written by Atsushi Kume, was originally published Online First without open access. After publication in volume 130, issue 3, page 501–514 the Botanical Society of Japan decided to opt for Open Choice and to make the article an open access publication. Therefore, the copyright of the article has been changed to © The Author(s) 2018 and the article is forthwith distributed under the terms of the Creative Commons Attribution 4.0 International License (http://creativecommons.org/licenses/by/4.0/), which permits use, duplication, adaptation, distribution and reproduction in any medium or format, as long as you give appropriate credit to the original author(s) and the source, provide a link to the Creative Commons license, and indicate if changes were made.

